# High Photocatalytic Performance of Two Types of Graphene Modified TiO_2_ Composite Photocatalysts

**DOI:** 10.1186/s11671-017-2224-4

**Published:** 2017-07-14

**Authors:** Jun Zhang, Sen Li, Bo Tang, Zhengwei Wang, Guojian Ji, Weiqiu Huang, Jinping Wang

**Affiliations:** 1 College of Energy and Power Engineering, Nanjing Institute of Technology, Nanjing city, 211167 China; 2 School of Petroleum Engineering, Changzhou University, Changzhou, 213016 People’s Republic of China

**Keywords:** Carbon materials, Energy storage and conversion, Solar energy materials

## Abstract

High quality and naturally continuous structure of three-dimensional graphene network (3DGN) endow it a promising candidate to modify TiO_2_. Although the resulting composite photocatalysts display outstanding performances, the lacking of active sites of the 3DGN not only goes against a close contact between the graphene basal plane and TiO_2_ nanoparticles (weaken electron transport ability) but also limits the efficient adsorption of pollutant molecules. Similar with surface functional groups of the reduced graphene oxide (RGO) nanosheets, surface defects of the 3DGN can act as the adsorption sites. However, the defect density of the 3DGN is difficult to control (a strict cool rate of substrate and a strict flow of precursor gas are necessary) because of its growth approach (chemical vapor deposition method). In this study, to give full play to the functions of graphene, the RGO nanosheets and 3DGN co-modified TiO_2_ composite photocatalysts are prepared. After optimizing the mass fraction of the RGO nanosheets in the composite photocatalyst, the resulting chemical adsorption ability and yields of strong oxidizing free radicals increase significantly, indicating the synergy of the RGO nanosheets and 3DGN.

**Electronic supplementary material:**

The online version of this article (doi:10.1186/s11671-017-2224-4) contains supplementary material, which is available to authorized users.

## Background

Because of its excellent electrical property and large BET area, graphene is considered as a promising modifier to improve the photocatalytic performance of TiO_2_ [1]. However, the high defect density and the discrete structure of the widely used reduction graphene oxide (RGO) nanosheets lead to the actual performances of the resulting photocatalysts are inferior to the theoretical predictions. With the development of research, three-dimensional graphene network (3DGN) has drawn increasing attention as a result of its naturally continuous structure and high quality, which are beneficial to enhancing the electron transport ability and loading ability (for TiO_2_ nanoparticles) [2, 3].

Recently, our group found that the defect density of graphene is closely related to the photocatalytic performance of the resulting 3DGN–TiO_2_ composite photocatalyst [2]. The core reason is that an optimizing amount of the surface defect not only provides enough chemisorption sites for pollutant molecules but also links the graphene basal plane and TiO_2_ nanoparticles closely to provide electron transport channels at their interface. However, controlling the defect density of the 3DGN during the chemical vapor deposition process is complex. Contrarily, surface functional groups of the RGO nanosheets, which possess the same functions, can be adjusted conveniently [4, 5]. Therefore, additional RGO nanosheets should bring about a better performance for the 3DGN–TiO_2_ photocatalyst.

In this study, the RGO–3DGN–TiO_2_ composite photocatalysts are prepared and optimized. The photocatalytic performances including the chemical adsorbability, the electron transport property, and phenol decomposition rate constants are studied, and the photoluminescence (PL), infrared (IR) spectrum, and electron paramagnetic resonance (EPR) spectrum are adopted to reveal the synergy between the 3DGN and RGO.

## Methods

The preparations of various photocatalysts and decomposition experiments have been described by our previous reports [2, 5, 6]. Briefly, the nickel foam with 3DGN was vertically immersed into 50 ml ammonia (25 wt%) solution with 50 mg TiO_2_–RGO nanosheets mixture (the mass fraction of the RGO are 1–8 wt%) at room temperature. Subsequently, the solution was transferred to an autoclave and heated up to 110 °C (keeping for 10 h) in the vacuum drying oven. The resulting photocatalyst was taken out after cooling down. Before the catalytic experiments, the photocatalyst was washed by deionized water and dried in the vacuum drying oven at 80 °C for 2 h.

## Results and Discussion

**Fig. 1:**
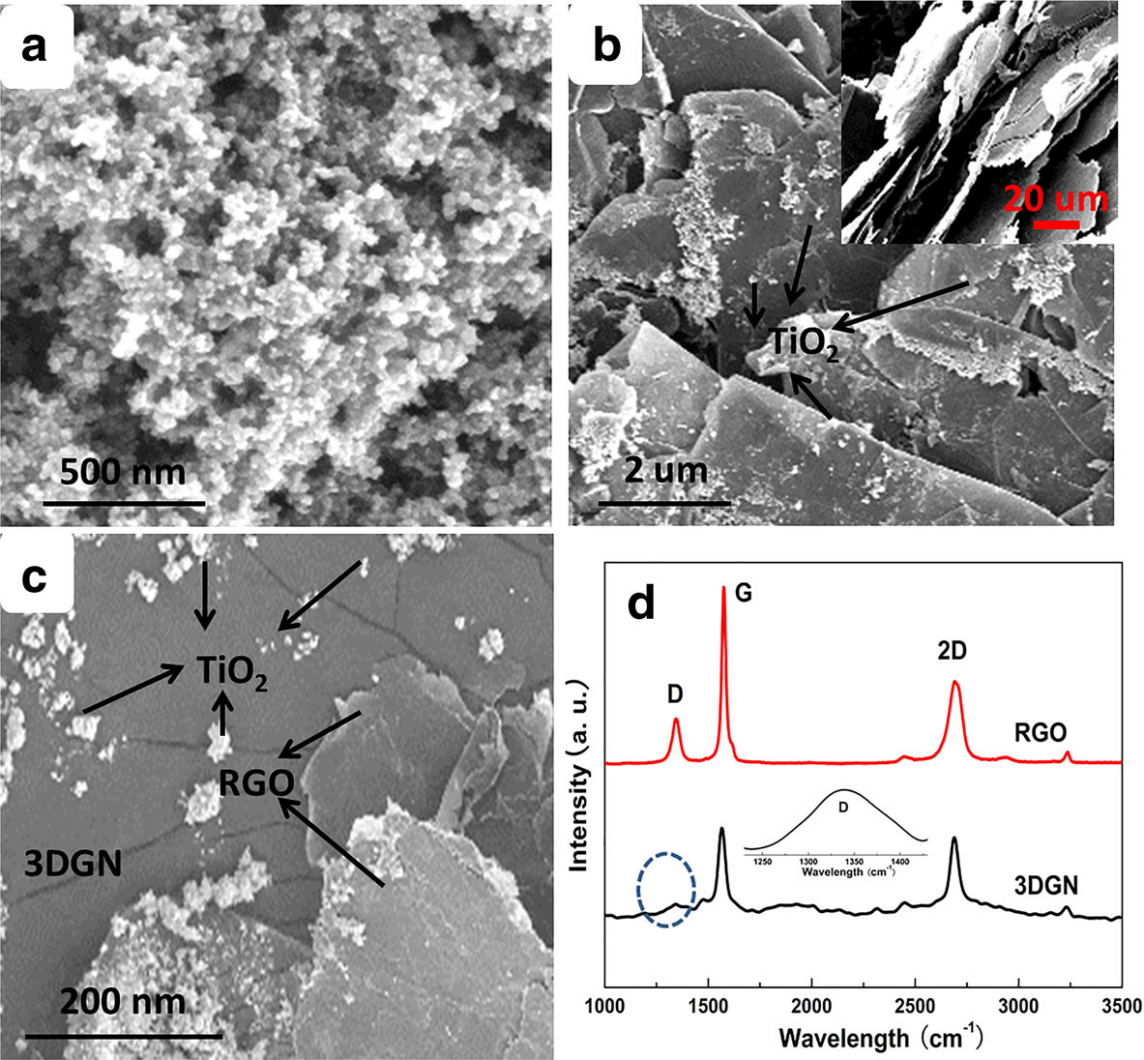
SEM images of the **a** pure TiO_2_ **b** 3DGN-TiO_2_, *inset* is the pristine 3DGN, **c** RGO–3DGN–TiO_2_, and **d** Raman curves of the RGO and 3DGN; the D peak of the 3DGN is magnified. The images **a**–**c** show SEM images of the pure TiO_2_, 3DGN–TiO_2_, and RGO–3DGN–TiO_2_. Therein, the *inset* of image **b** is the SEM image of origin 3DGN. Figure **d** display Raman curves of the RGO and 3DGN, the D peak of the 3DGN is magnified. The obvious wrinkle on surface of the 3DGN, which is closely related to its adsorption ability (for pollutants) and loading capacity (for TiO_2_), is caused by the distinction between thermal expansion coefficients of graphene and Ni substrate

SEM images of the pure TiO_2_ and 3DGN–TiO_2_ are shown in Fig. 1a, b, and the pristine 3DGN is displayed in the inset. The obvious wrinkle on surface of the 3DGN, which is closely related to its adsorption ability (for pollutant molecules) and loading capacity (for TiO_2_ nanosheets), is caused by the distinction between thermal expansion coefficients of graphene and Ni substrate. Comparing with that of the 3DGN–TiO_2_, the RGO–3DGN–TiO_2_ photocatalyst displays a similar appearance (Fig. 1c, SEM image), and the average size of TiO_2_ particles ranges from 10 to 50 nm, indicating the excessive agglomeration can be avoided by utilizing the large BET area of the 3DGN (Table S1 of Additional file 1) [1, 2]. In order to give full play to its advantage of the RGO, the sample quality is optimized, which is confirmed by the low intensity of the D peak from Raman curve (*I*_D_/*I*_G_ = 0.29, Fig. 1d) [7]. Based on the recent founding from our group, the presence of a moderate defect density of the 3DGN is in favor of the high performance of the resulting composite photocatalysts. Therefore, an inconspicuous D peak can be seen from Raman profile of the adopted 3DGN because of the well-designed defect density [6].

**Fig. 2:**
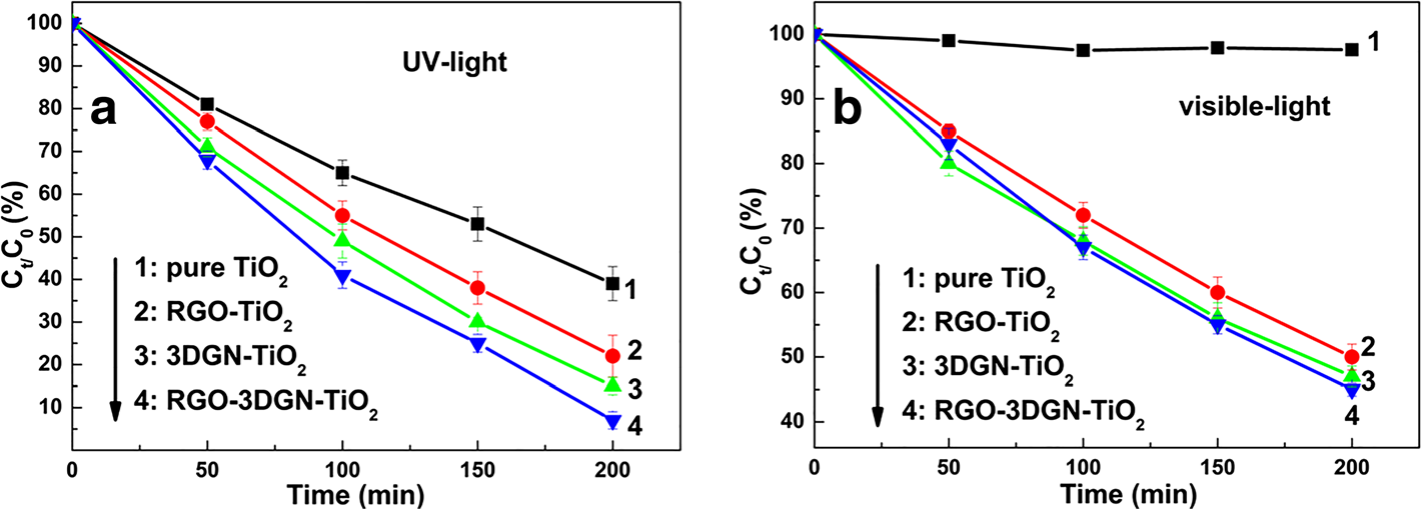
Decomposition experiments of phenol under **a** UV-light and **b** visible-light irradiation

**Fig. 3:**
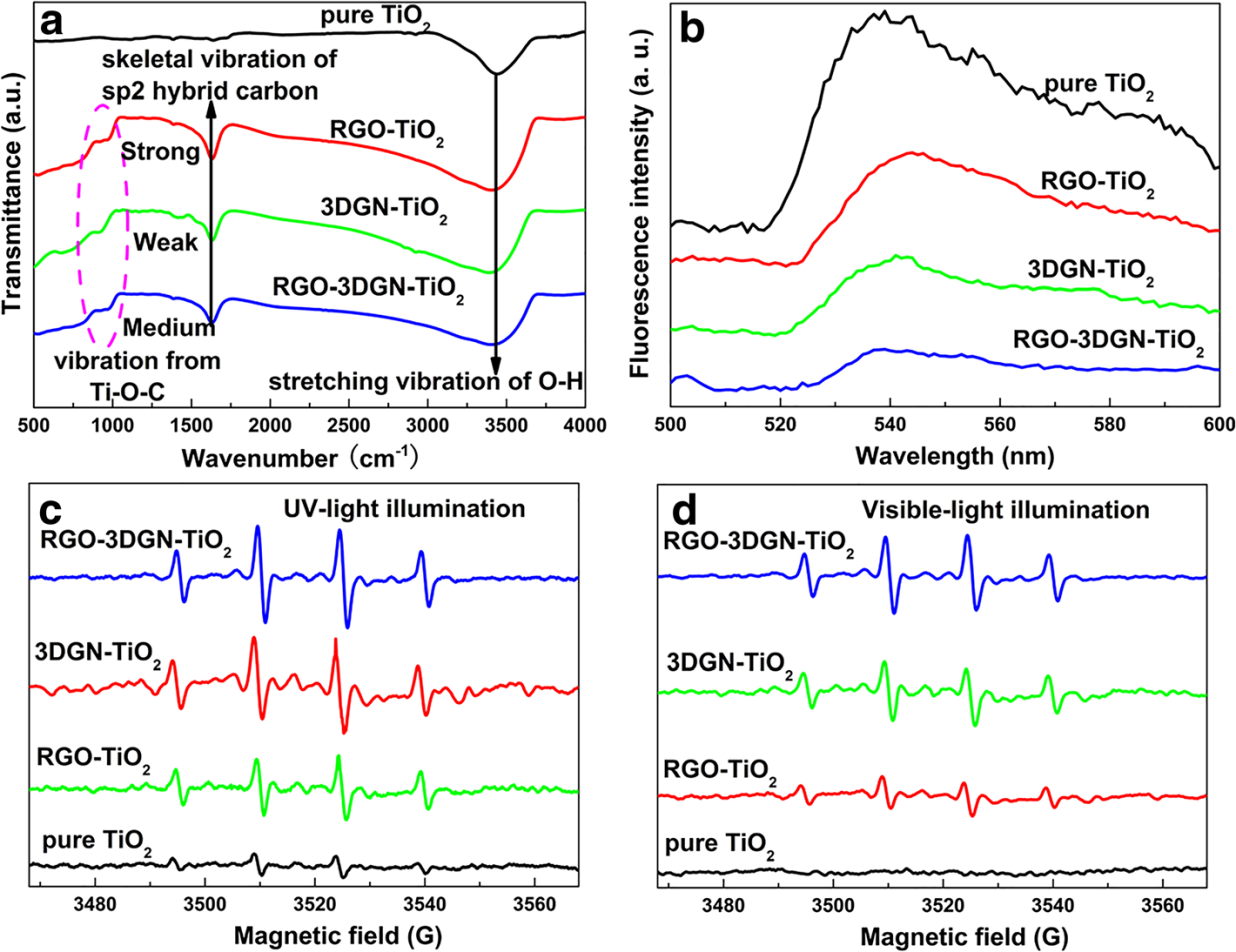
Characterizations of various composite photocatalysts. **a** IR curves and **b** PL patterns of various photocatalyst, EPR spectra of radical adduct trapped by 5,5-dimethyl-1-pyrroline-*N*-oxide under **c** UV-light and **d** visible-light irradiation

Photocatalytic performance of the RGO–3DGN–TiO_2_ composite photocatalyst is evaluated by phenol decomposition experiments. The decomposition rate constant of phenol under UV-light irradiation is as high as 1.33 × 10^−2^ min^−1^, which is 180, 70, and 40% higher than those cases of using the pure TiO_2_, RGO–TiO_2_, and 3DGN–TiO_2_, respectively (Fig. 2a, eight parallel experiments have been performed for each decomposition test to ensure the repeatability; the error bar is supplied). Similarly, the resulting composite photocatalyst displays excellent performance under visible-light illumination (Fig. 2b). Two key factors, utilization rate of the photo-induced electrons and chemisorption amount of pollutants, of the prepared composite photocatalysts determine their photocatalytic property under UV-light irradiation. In the theory, the relatively large BET area and high quality of the 3DGN (compared with the RGO) endow it an outstanding electron tank to achieve the separation of the photo-generated electron–hole pairs and an excellent carrier to adsorb more pollutants. However, the actual performance is inferior to the expectation due to the unsatisfied contact between the graphene basal plane and TiO_2_ (lacking electron transport channels at their interface). Moreover, the adsorption amount of pollutants is limited because of insufficient active adsorption sites on the 3DGN surface (the interaction between the high-quality graphene basal plane and pollutant molecules is weak π–π interaction (or Van der Waal force) rather than the strong chemical bond). Contrarily, the surface functional groups of the RGO provide plentiful active sites to chemisorb pollutants. Adsorbabilities of these composites are listed in Additional file 1: Table S2, and the RGO–3DGN–TiO_2_ with the optimized RGO nanosheets (including the mass fraction and surface functional groups amount) shows the highest chemisorption amount of pollutants although its BET area almost equals that of the 3DGN–TiO_2_. On the other hand, the addition of RGO nanosheets achieves a closely contact between the graphene basal plane and TiO_2_, which can be proved by the IR spectrum. As shown in Fig. 3a, the wide absorption peak at high-frequency area of TiO_2_ is induced by the O–H stretching vibration of the surface hydroxyl from adsorbed water, while the low frequency adsorption below 1000 cm^−1^ is attributed to the Ti–O–Ti vibration [5]. The ~1600 cm^−1^ signal of the composite photocatalyst is assigned to the skeletal vibration of graphene sheets [8]. After comparing the profiles of the RGO–3DGN–TiO_2_ and 3DGN–TiO_2_, a change in the intensity at 800 cm^−1^, the signal of the Ti–O–C vibration, can be seen, indicating the enhanced chemical bond between the graphene basal plane and TiO_2_ after adding the RGO nanosheets [2, 5].

**Fig. 4:**
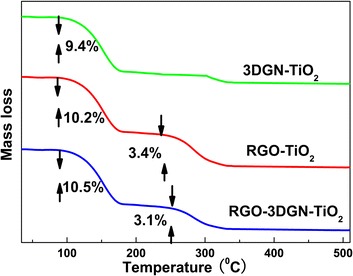
TGA curves of the 3DGN–TiO_2_, RGO–TiO_2_, and RGO–3DGN–TiO_2_

Under visible-light irradiation, the function of graphene in the photocatalysts is sensitizer, and the electron transport channels between graphene and TiO_2_ also act as a vital role for the resulting photocatalytic performance. The decomposition rate constants of phenol by using the 3DGN–TiO_2_ and RGO–3DGN–TiO_2_ are similar; manifesting the additional RGO nanosheets does not give rise to a remarkable effect under visible-light irradiation. The possible reason is that the electron transport from graphene to TiO_2_ (quantum tunneling) is difficult to further enhance by adding the RGO nanosheets because of their uncontrollable thickness (the tunneling probability of the photo-induced electrons dependent on the graphene thickness) [5]. Moreover, it is worth noting that the relatively high defect density and discontinuous structure of the RGO nanosheets go against the long lifetime of the photo-induced electrons. Therefore, the adding amount and reduction degree of the RGO nanosheets must be optimized to achieve the synergy between the RGO and 3DGN (more optimizing details are shown in Table S3 in Additional file 1). Moreover, the TGA tests were carried out to provide more information on the resulting composite photocatalysts (Fig. 4). As for the 3DGN–TiO_2_ sample, a remarkable weight loss stage can be seen in the temperature range 100–180 °C, which is caused by the evaporation of adsorbed water on the surface. On the other hand, an additional weight loss stage at 250–350 °C can be found for the RGO (8 wt%)–TiO_2_ and RGO (8 wt%)–3DGN–TiO_2_ photocatalysts, and the similar weight loss ratios of them indicate the identical source (the remove of residual surface functional groups of the RGO nanosheets).

The PL curves of various photocatalysts under UV-light irradiation are shown in Fig. 3b. The signal resulted from the radiative recombination of the self-trapped excitons in TiO_2_ remarkably reduces for the composite photocatalysts, manifesting the depressed recombination of electron–hole pairs. Therein, the highest utilization rate of the photo-induced electrons (compared with that of other two composites) is achieved in the RGO–3DGN–TiO_2_, which is confirmed by its weakest signals. The fundamental reason is that surface functional groups of the RGO nanosheets provide a bridge to link the graphene basal plane and TiO_2_, enhancing the electron transport ability from TiO_2_ to 3DGN. A synergy can be achieved when an additional 2 wt% RGO nanosheets is added.

The EPR curves of various samples under UV-light irradiation are shown in the Fig. 3c. The yields of ${\mathrm{OH}}^{\cdotp }$ and ${\displaystyle {0}_2^{-}}$ (the active substances to decompose pollutants) directly determine the resulting photocatalytic performance. The stronger signals from the RGO–3DGN–TiO_2_ photocatalyst indicates that the added RGO nanosheets actually promote the electron transport at the interface (prolong electron lifetime) under UV-light irradiation. As for the case of visible-light activity, the 3DGN–TiO_2_ and RGO–3DGN–TiO_2_ display similar signal intensity (Fig. 3d), which is consistent with the decomposition experiments. Under visible-light irradiation, the source of the photo-generated electron is graphene, and the electrons which can react with dissolved oxygen molecules in solution to produce 0H and ${\displaystyle {0}_2^{-}}$ must conquer the Schottky barrier at the interface to inject into TiO_2_ [5]. Although the surface functional groups of the RGO nanosheets act as a bridge to enhance the quantum tunneling behavior (a pre-condition for the *π–d* electron coupling between graphene and TiO_2_), the uncontrolled thickness of RGO nanosheets exert a negative effect for the tunneling probability because the width of the Schottky barrier is determined by the thickness of graphene [5]. Therefore, the added RGO nanosheets do not induce a prominent improvement to the observed visible-light activity.

## Conclusions

The RGO nanosheets and 3DGN co-modified TiO_2_ composite photocatalysts were prepared to improve the photocatalytic performance. Although the discontinuous structure and high defect density of the RGO nanosheets may shorten the lifetime of the photo-induced electrons, their surface functional groups impose a positive effect to the chemisorption ability for pollutants and the electron transport capability between the graphene basal plane and TiO_2_, which avoids the complex adjusting process to control the defect density of the 3DGN. The decomposition rate constant of phenol reaches 1.33 × 10^−2^ min^−1^ under UV-light irradiation after achieving the synergy between the RGO nanosheets and 3DGN, which is much higher than those cases of the RGO–TiO_2_ and 3DGN–TiO_2_ photocatalysts.

## Additional files

Additional file 1: Table S1. BET area of the pure TiO_2_ and composite photocatalysts. **Table S2.** Adsorption abilities of the RGO–3DGN–TiO_2_ and 3DGN–TiO_2_ under various temperatures, residual amount of pollutants are listed. **Table S3.** The relationship between mass fraction (and reduction degree) of the RGO nanosheets in the RGO–3DGN–TiO_2_ photocatalyst and decomposition rate constant of phenol. (DOC 196 kb)
