# Supplementary material for: High Photocatalytic Performance of Two Types of Graphene Modified TiO2 Composite Photocatalysts
Source: Nanoscale Res Lett. 2017 Jul 14;12:457. doi: 10.1186/s11671-017-2224-4 (PMC5511128; doi:10.1186/s11671-017-2224-4)
Supplement: Supplementary file 1 — BET area of the pure TiO2 and composite photocatalysts. Table S2. Adsorption abilities of the RGO–3DGN–TiO2 and 3DGN–TiO2 under various temperatures, residual amount of pollutants are listed. Table S3. The relationship between mass fraction (and reduction degree) of the RGO nanosheets in the RGO–3DGN–TiO2 photocatalyst and decomposition rate constant of phenol. (DOC 196 kb) [file 11671_2017_2224_MOESM1_ESM.doc]

**High photocatalytic performance of two types of graphene modified TiO_2_ composite photocatalysts**

Jun Zhang^1^, Sen Li^2^, Bo Tang^2^*, Zhengwei, Wang^2^, Guojian Ji^2^, Weiqiu, Huang^2^*, Jinping Wang^1^

*1. College of Energy and Power Engineering,* *Nanjing Institute of Technology, Nanjing city, 211167, China*

*2. School of Petroleum Engineering, Changzhou University, Changzhou 213016, People’s Republic of China*

**1. Experimental**

**1.1. Materials and chemicals**

Nanoscale TiO_2_ was purchased from Shanghai Jianghu industrial Co., Ltd. Deionized water (resistivity 18 Mcm) was utilized to preparation all the solution. Nickel foam (with areal density 300 gm^-2^ and thickness 10 mm) was purchased from Haobo Co., Ltd. (Shenzhen, China). Phenol and ammonia were obtained commercially from the Beijing chemical reagent plant (Beijing, China).

**1.2 Preparation**

The detailed preparations of 3DGN, RGO, RGO-TiO_2_ and 3DGN-TiO_2_ have been reported in our previous work [1-5]. The RGO-3DGNs-TiO_2_ composite photocatalyst was synthesized by hydrothermal method. Briefly, the nickel foam with 3DGN was vertically immersed into 50ml ammonia (25 wt%) solution with 50mg TiO_2_-RGO nanosheets mixture (the mass fraction of RGO ranges from 1-8 wt% ) at room temperature. Subsequently, the solution was transferred to an autoclave and heated up to 110°C for 10h in the vacuum drying oven. The resulting photocatalyst was taken out after cooling down natural. Before the catalytic experiments, the catalyst was washed with deionized water and dried in the vacuum drying oven at 80 °C for 2 h.

**1.3 Characterizations**

Scanning electron microscope (SEM) images were obtained by FEI Sirion 200 scanning electron microscope working at 5 kV. Raman spectra were performed by LabRam-1B Raman microspectrometer at 514.5 nm (Horiba Jobin Yvon, France). The Photoluminescence (PL) spectra were measured on QM4CW (Photo Technology International). Electron Paramagnetic Resonance (EPR) results were recorded on EPR-8 (Bruker BioSpin Corp., Germany). The instrumental settings are listed as following: center field 3480.00 Gauss, modulation frequency 100 kHz, modulation amplitude 2 G, microwave frequency at 9.74 GHz, and microwave power is 7mW. The presence of superoxide and hydroxyl radicals can be trapped by 5,5-dimethyl-1-pyrroline-N-oxide (DMPO) and show corresponding signals in ERS spectrum. Fourier transform infrared spectroscopy (FTIR) curves were measured on IR Prestige-21 system (PerkinElmer). Thermogravimetric analysis (TGA) was measured with a Pyris I TGA instrument (PerkinElmer, U.S.A.).

**1.4 Photocatalyst measurements**

The photocatalytic reaction system contains a 500 W xenon lamp and a cutoff filter (an enclosed vessel was filled with 1 molL^-1^ NaNO_2_ solution, and the cut-off wavelength is 400 nm). Photocatalytic activities of the catalysts were evaluated by degradation of phenol. In a typical process, photocatalysts were horizontally immersed into 50 mL phenol solution (60 mgL^-1^) and then irradiated under xenon lamp, and 2 mL solution was sampled for analysis at certain time intervals.

**2. Results and discussion**

**2.1 OH^●^ radical measurement**

The presence of the OH^●^ radical was detected by PL spectra with terephthalic acid (purchased from the Beijing chemical reagent plant, China) as a probe molecule. 2-Hydroxyl-terephthalic acid, as a strong fluorescence material, would form after the terephthalic acid capturing the OH^●^ radicals. Under visible-light illumination, obvious peak belonged to the 2-Hydroxyl-terephthalic acid can be seen with various composite photocatalysts (Fig. S1, 80ml terephthalic acid aqueous solutions, 5mmol/L), and the intensity of signal display the yield of the OH^●^ and corresponding photocatalytic activities of various photocatalysts.


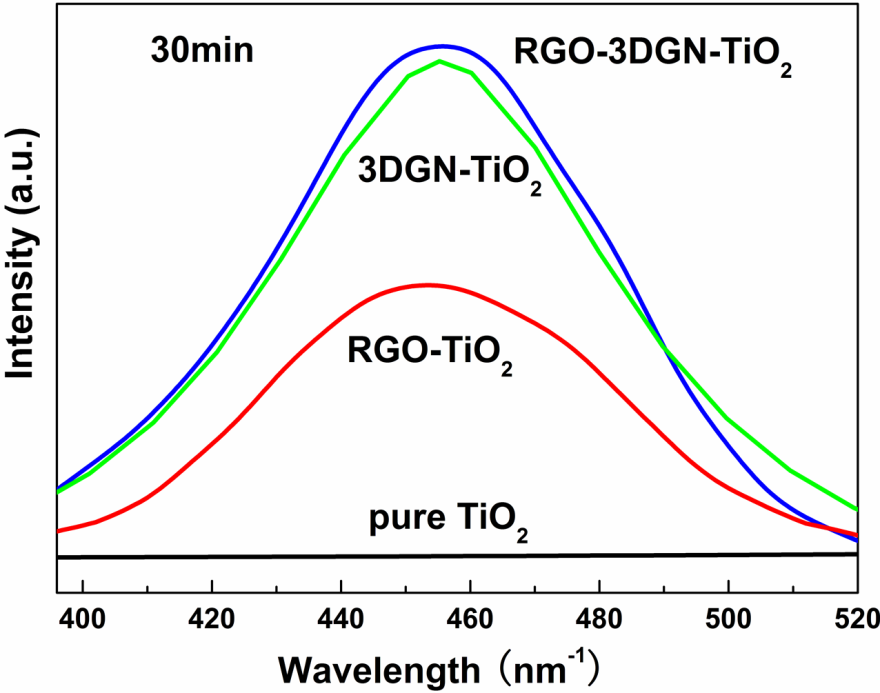


Figure S1 OH^●^–trapping PL spectra by using various photocatalysts under visible-light illumination.

**2.2 Chemisorption ability**

Based on the residual concentration of phenol in aqueous solution at room temperature, RGO-3DGN-TiO_2_ and 3DGN-TiO_2_ show similar adsorption ability, indicating BET area is the determinant for their adsorption ability. However, both the physical adsorbed pollutants and chemical adsorbed pollutants are included, while only the latter contributes to the resulting photocatalytic performance. In order to estimate the ratio of chemical adsorbed part, the corresponding adsorption tests of them under various temperatures are performed. With increased temperature, physical adsorption is depressed (Van der Waals' force is difficult to bound pollutant molecules due to their increased average kinetic energy). Therefore, the adsorption amount of pollutants under high temperature can be approximately considered as the chemical adsorption, which is closely related to the reducing degree of the RGO nanosheets (Table S2, the photocatalyst with 2 wt% RGO (and 4h reduction time) is found the optimum value).

**2.3 Relationship between functional group amount of the RGO nanosheets and photocatalytic performance**

The major functions of the added RGO nanosheets include improving chemisorption ability for pollutants and promoting electron transport at the interface between graphene basal plane and TiO_2_, which are closely related to the reducing degree of the adopted RGO nanosheets (residual amount of surface functional groups). The corresponding decomposition rate constants of phenol are listed in the Table S3. The sample with 2 wt% (and 4h reduction time) displays the best performance, which is in line with the chemisorption ability.

Table S1 BET area of the pure TiO_2_ and composite photocatalysts.

| Samples | Pure TiO_2_ | RGO-TiO_2_ | 3DGN-TiO_2_ | RGO(2wt%)-3DGN-TiO_2_ |
| --- | --- | --- | --- | --- |
| BET area (m^2^g^-1^) | 48.6 | 163.4 | 475.3 | 456.6 |

Table S2 Adsorption abilities of the RGO-3DGN-TiO_2_ and 3DGN-TiO_2_ under various temperatures, residual amount of pollutants are listed.

| Samples | Reduction time of RGO  (hour) | Residual amount of phenol | | |
| --- | --- | --- | --- | --- |
|  |  | 20^0^C | 50^0^C | 80^0^C |
| 3DGN-TiO_2_ | -- | 73% | 84% | 90% |
| RGO (1 wt%)-3DGN-TiO_2_ | 4 | 73% | 82% | 88% |
| RGO (2 wt%)-3DGN-TiO_2_ | 1 | 73% | 78% | 84% |
| RGO (2 wt%)-3DGN-TiO_2_ | 4 | 73% | 81% | 86% |
| RGO (2 wt%)-3DGN-TiO_2_ | 8 | 73% | 83% | 90% |
| RGO (5 wt%)-3DGN-TiO_2_ | 4 | 74% | 79% | 84% |
| RGO (5 wt%)-3DGN-TiO_2_ | 8 | 73% | 83% | 89% |
| RGO (8 wt%)-3DGN-TiO_2_ | 4 | 73% | 78% | 81% |

Table S3 The relationship between mass fraction (and reduction degree) of the RGO nanosheets in the RGO-3DGN-TiO_2_ photocatalyst and decomposition rate constant of phenol.

| Samples | Reduction time of RGO (hour) | Decomposition rate constant of phenol (min^-1^) | |
| --- | --- | --- | --- |
|  |  | UV-light irradiation | Visible-light irradiation |
| Pure TiO_2_ | -- | (4.71±0.31)×10^-3^ | ~0 |
| RGO (5 wt%)-TiO_2_ | 4 | (7.58±0.42)×10^-3^ | (3.46±0.20)×10^-3^ |
| 3DGN-TiO_2_ | -- | (9.48±0.38)×10^-3^ | (3.78±0.16)×10^-3^ |
| RGO (1 wt%)-3DGN-TiO_2_ | 4 | (9.85±0.28)×10^-3^ | (3.89±0.22)×10^-3^ |
| RGO (2 wt%)-3DGN-TiO_2_ | 1 | (1.09±0.15)×10^-2^ | (3.85±0.19)×10^-3^ |
| RGO (2 wt%)-3DGN-TiO_2_ | 4 | (1.33±0.13)×10^-2^ | (4.00±0.17)×10^-3^ |
| RGO (2 wt%)-3DGN-TiO_2_ | 8 | (9.90±0.33)×10^-3^ | (3.75±0.28)×10^-3^ |
| RGO (5 wt%)-3DGN-TiO_2_ | 4 | (1.14±0.14)×10^-2^ | (3.74±0.21)×10^-3^ |
| RGO (5 wt%)-3DGN-TiO_2_ | 8 | (8.17±0.22)×10^-3^ | (3.36±0.36)×10^-3^ |
| RGO (8 wt%)-3DGN-TiO_2_ | 4 | (8.76±0.39)×10^-3^ | (3.51±0.11)×10^-3^ |

**References**

[1] B. Tang, G. X. Hu. Chem Vapor Deposit 20, 2014, 14-22.

[2] B. Tang, G. X. Hu, H. Y. Gao et al. J Power Source 2013, 234, 60-68.

[3] Y. F. Sun, Y. C. Cao, W. Q. Huang. Mater Letter 165, 2016, 178-180.

[4] Y. F. Sun, X. B. Wang, B. Tang et al. Mater Lett 189, 2017, 54–57.

[5] G.X. Hu, B. Tang, Mater Chem Phys 138 (2013) 608–614.
